# Combining tDCS With a Motor-Cognitive Task to Reduce the Negative Impact of Dual-Tasking on the Gait of Older Adults

**DOI:** 10.1093/geroni/igaa057.921

**Published:** 2020-12-16

**Authors:** Jeffrey Hausdorff, Nofar Schneider, Marina Brozgol, Pablo Cornejo Thumm, Nir Giladi, Rachel Katz, Anat Mirelman, Brad Manor

**Affiliations:** 1 Tel Aviv Sourasky Medical Center, Tel Aviv, Tel Aviv, Israel; 2 Tel Aviv University, Tel Aviv, Israel; 3 Hebrew SeniorLife/Harvard Medical School, Boston, Massachusetts, United States

## Abstract

The simultaneous performance of a secondary task while walking (i.e., dual tasking) increases motor-cognitive interference and fall risk in older adults. Combining transcranial direct current stimulation (tDCS) with the concurrent performance of a task that putatively involves the same brain networks targeted by the tDCS may reduce the negative impact of dual-tasking on walking. We examined whether tDCS applied while walking reduces the dual-task costs to gait and whether this combination is better than tDCS alone or walking alone (with sham stimulation). In 25 healthy older adults (aged 75.7±10.5yrs), a double-blind, within-subject, cross-over pilot study evaluated the acute after-effects of 20 minutes of tDCS targeting the primary motor cortex and the dorsal lateral pre frontal cortex during three separate sessions:1) tDCS while walking on a treadmill in a virtual-reality environment (tDCS+walking), 2) tDCS while seated (tDCS+seated), and 3) walking in the virtual-reality environment with sham tDCS (sham+walking). The complex walking condition taxed motor and cognitive abilities. During each session, single- and dual-task walking and cognitive function were assessed before and immediately after stimulation. Compared to pre-tDCS performance, tDCS+walking reduced the dual-task cost to gait speed (p=0.004) and other gait features (e.g., variability p=0.02), and improved (p<0.001) executive function (Stroop interference score). tDCS+seated and sham+walking did not affect the dual-task cost to gait speed (p>0.17). These initial findings demonstrate that tDCS delivered during challenging walking ameliorates dual-task gait and executive function in older adults, suggesting that the concurrent performance of related tasks enhances the efficacy of the neural stimulation and mobility.

